# Comprehensive Overview of *Toxoplasma gondii*-Induced and Associated Diseases

**DOI:** 10.3390/pathogens10111351

**Published:** 2021-10-20

**Authors:** Darine Daher, Ahmad Shaghlil, Eyad Sobh, Maguy Hamie, Malika Elhage Hassan, Mohamad Bahij Moumneh, Shaymaa Itani, Rana El Hajj, Lina Tawk, Marwan El Sabban, Hiba El Hajj

**Affiliations:** 1 Department of Experimental Pathology, Microbiology and Immunology, Faculty of Medicine, American University of Beirut, Beirut 1107 2020, Lebanon; dkd04@mail.aub.edu (D.D.); mh242@aub.edu.lb (M.H.); mae87@mail.aub.edu (M.E.H.); mmm106@mail.aub.edu (M.B.M.); ski02@mail.aub.edu (S.I.); 2 Department of Biology, Faculty of Sciences, R. Hariri Campus, Lebanese University, Beirut 1107 2020, Lebanon; ahmad.shaghlil.98@gmail.com (A.S.); eyadsobh18@gmail.com (E.S.); 3 Department of Biological Sciences, Beirut Arab University, Beirut 1107 2809, Lebanon; r.hajj@bau.edu.lb; 4 Department of Medical Laboratory Sciences, Faculty of Health Sciences, University of Balamand, Beirut 1100 2807, Lebanon; lina.tawk@balamand.edu.lb; 5 Department of Anatomy, Cell Biology and Physiological Sciences, Faculty of Medicine, American University of Beirut, Beirut 1107 2020, Lebanon; me00@aub.edu.lb

**Keywords:** toxoplasmosis, neuropathies, psychiatric disorders, behavioral disorders, neurological disorders

## Abstract

*Toxoplasma gondii* (*T. gondii*) is a prevalent protozoan parasite of medical and veterinary significance. It is the etiologic agent of toxoplasmosis, a neglected disease in which incidence and symptoms differ between patients and regions. In immunocompetent patients, toxoplasmosis manifests as acute and chronic forms. Acute toxoplasmosis presents as mild or asymptomatic disease that evolves, under the host immune response, into a persistent chronic disease in healthy individuals. Chronic toxoplasmosis establishes as latent tissue cysts in the brain and skeletal muscles. In immunocompromised patients, chronic toxoplasmosis may reactivate, leading to a potentially life-threatening condition. Recently, the association between toxoplasmosis and various diseases has been shown. These span primary neuropathies, behavioral and psychiatric disorders, and different types of cancer. Currently, a direct pre-clinical or clinical molecular connotation between toxoplasmosis and most of its associated diseases remains poorly understood. In this review, we provide a comprehensive overview on *Toxoplasma*-induced and associated diseases with a focus on available knowledge of the molecular players dictating these associations. We will also abridge the existing therapeutic options of toxoplasmosis and highlight the current gaps to explore the implications of toxoplasmosis on its associated diseases to advance treatment modalities.

## 1. Introduction

*Toxoplasma gondii* (*T. gondii*) is an obligate intracellular parasite that infects a broad range of animals including approximately one third of the world’s human population [1]. The prevalence of *T. gondii* infection varies widely between countries [2]. In North America, South East Asia, and Northern Europe, prevalence is low and ranges between 10 and 30%. In Central and Southern Europe, a moderate prevalence is reported and ranges between 30 and 50%, while in Latin America and tropical African countries, a high prevalence is common and reaches an alarming percentage of 80% in certain regions [2]. The Center for Disease Control and Prevention (CDC) reported that more than 40 million people in the United States are infected with this parasite, and classified toxoplasmosis among the neglected parasitic infections requiring public health action control [3].

The *T. gondii* life cycle involves a sexual stage occurring in the intestinal epithelium of felines and an asexual part involving any warm-blooded animal. It exhibits three morphologically distinct infectious stages: tachyzoites (responsible for acute toxoplasmosis leading to tissue damage), bradyzoites (responsible for chronic toxoplasmosis manifested as cysts in the brain and skeletal muscle tissues), and sporozoites (infective forms found in oocysts shed in cats’ feces). Human infection starts following the oral ingestion of sporulated oocysts in food or water contaminated with felines’ feces, or upon the ingestion of tissue cysts after the consumption of contaminated raw or undercooked meat. Vertical transmission follows the transplacental spread of tachyzoites from a primo-infected pregnant mother to her fetus/baby, leading to congenital toxoplasmosis.

## 2. *Toxoplasma gondii* Pathogenesis

**Figure 1:**
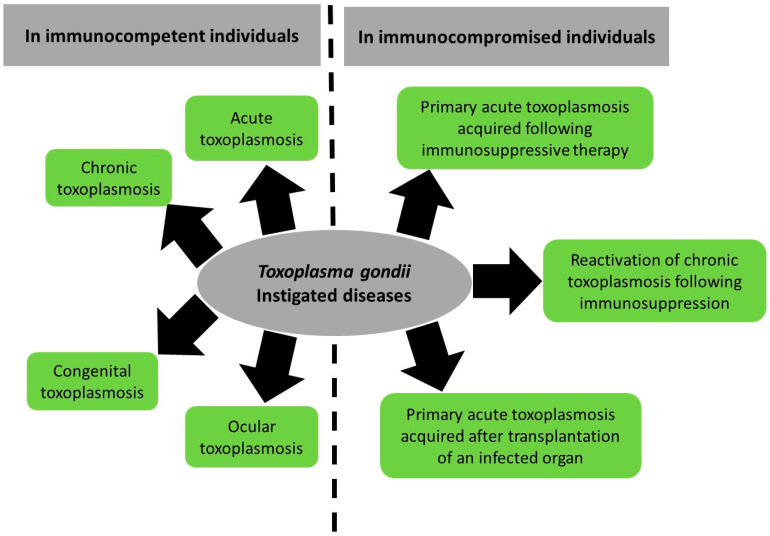
Summary of *Toxoplasma gondii*-induced diseases and their spectrum between immunocompetent and immunocompromised patients.

The manifestations of toxoplasmosis differ between patients (Figure 1). In the sections below, we will provide an overview on the direct pathogenesis of *T. gondii* spanning acute, chronic, congenital, and ocular infection and reactivated chronic toxoplasmosis. We will also summarize the reported literature on *T. gondii*-associated diseases.

### 2.1. Toxoplasmosis in Immunocompetent Patients

#### 2.1.1. Acute Toxoplasmosis

Acute toxoplasmosis develops after an incubation period of a few days following tachyzoites’ spread and replication. It is asymptomatic in more than 80% of immunocompetent individuals [4,5]. It can manifest with flu-like symptoms including fever and mononucleosis-like symptoms, with cervical posterior adenopathy, myalgia, and asthenia [4]. Occasionally, chorioretinitis may occur. The severity of infection is also related to the genotype of the parasite strain. In North America and Europe, six genetic markers were used to group *T. gondii* strains into clonal lineage I, II, and III, with I considered to have the highest virulence in preclinical mouse models, II less virulence, and III considered to be avirulent [6]. In French Guiana and Latin America, atypical strains showed high genetic diversity and represented a severe acquired toxoplasmosis among immunocompetent individuals. These subjects developed fatal pneumonitis, myocarditis, meningo-encephalitis, and polymyositis [2]. Tachyzoites disseminate to the brain and the skeletal muscles, and after the onset of the host immune system, they convert into bradyzoite cysts, initiating the chronic form of the disease. To enter the CNS, three mechanisms have been proposed: the “Trojan horse” mechanism, through which the parasite highjacks an immune cell to enter, the paracellular crossing mechanism, and the transcellular crossing mechanism [7]. The “Trojan horse” mechanism was based on several in vitro studies showing that infected immune cells exhibit increased motility and are capable of crossing endothelial barriers [8,9,10]. Furthermore, intravenous inoculation of mice with infected macrophages or dendritic cells resulted in the hastened appearance of the parasites in the CNS when compared to the inoculation of mice with free tachyzoites [9,11]. In the paracellular mechanism of entry, it has been proposed that *T. gondii* uses its actin-myosin motors, hence gliding motility, to cross the BBB [12].

#### 2.1.2. Congenital Toxoplasmosis

In sero-negative pregnant women, primary infection with *T. gondii* occurs following the placental transmission of the parasite to the fetus [13]. The degree of severity of congenital toxoplasmosis is inversely related to the gestational trimester at which the infection is acquired [14]. Although the placenta represents a major forefront that inhibits tachyzoites’ transmission in the beginning of gestation, this ability decreases gradually throughout the pregnancy, allowing the tachyzoites to move between cells and infect the fetus [15]. It is estimated that about 25% of *T. gondii* transmission takes place in the first trimester, whereas 54% and 65% of transmission occur in the second and third trimesters, respectively [16]. Infection of the fetus during the first trimester often leads to abortion, stillbirth, or a child born with severe abnormalities of the brain and eyes, such as hydrocephalus, intracranial calcifications, deafness, mental retardation, seizures, retinochoroiditis, and even blindness (reviewed in [17]). Transmission to the fetus in the second or third trimester is less likely to cause such severe clinical manifestations, but may result in subclinical disease, which may lead to retinochoroiditis or learning difficulties after birth [18]. It is worth noting that the percentage of acquiring toxoplasmosis during pregnancy varies according to regions and prevalence [19,20], and re-infection with atypical *T. gondii* genotypes was reported even in sero-positive pregnant women [21], and resulted in a more severe congenital toxoplasmosis [22].

#### 2.1.3. Ocular Toxoplasmosis

*T. gondii* is one of the primary causes of infectious uveitis worldwide, typically presenting with retinochoroiditis [23]. Ocular toxoplasmosis mostly occurs after an acquired congenital toxoplasmosis. Yet, some studies reveal postnatal acquired infections leading to this manifestation [24]. Clinical features of ocular toxoplasmosis depend on the anatomical location of the lesion [25]. Typically, retinochoroiditis is the most predominant indication of active intraocular inflammation. It presents with posterior uveitis, vitritis, focal necrotizing granulomatous retinitis, and reactive granulomatous choroiditis [26]. The rupture of intra-retinal cysts may lead to the reactivation of ocular toxoplasmosis, triggering a rapid localized immune reaction involving mostly Interleukin-17A [27]. 

#### 2.1.4. Chronic Toxoplasmosis

*T. gondii* can be classified as a primarily neurotropic pathogen, having a higher affinity for the central nervous system over other organs (reviewed in [28]). To reach the brain parenchyma from the cerebral blood circulation, different strains of *T. gondii* cross the brain endothelium to the capillary bedding through either hijacking leukocytes or as free parasites [7]. Once the blood brain barrier is crossed, the host immune response, among other factors including intracellular neuronal homeostasis, is triggered, and consequently, *T. gondii* tachyzoites switch to forming bradyzoite cysts, which are the hallmark of the chronic phase of the infection. These intraneuronal cysts are controlled but not eliminated by the immune system (reviewed in [29,30]). Although bradyzoites are slowly replicating forms, their replication affects the structure of the neurons and disrupt their connection. Furthermore, these tissue cysts trigger a brain-specific immune response (reviewed in [30]). Brain-resident cells including astrocytes, microglia, and neurons contribute to the intracerebral immune response by the production of cytokines, chemokines, and the expression of immune-regulatory cell surface molecules, such as major histocompatibility (MHC) antigens (reviewed in [29,30]). Circulating immune cells are also recruited to the site of infection in the CNS and contribute to the response against the infection [31,32]. The release of different cytokines, such as interleukins and interferons, including IL12, IL-1β, IL-6, and iNOS, among others, in addition to tumor necrosis factor-alpha (TNF-α) and interferon-gamma (IFN-γ), which activates IFN-inducible GTPases, is important for the cell-autonomous immunity and is effective to inhibit *T. gondii* replication ([33,34,35,36,37], reviewed in [31]). Infiltrating CD4^+^ and CD8^+^ T cells release several cytokines, mainly IFN- γ (reviewed in [30]). The immune response causes brain inflammation, which leads to ventricular dilatation, disrupting neuronal structure and connectivity [38,39,40]. Morphological changes include altered fiber density, loss of fiber continuity, a reduction in postsynaptic density protein 95 (PSD95) and synaptophysin, and synaptic proteins. The reduction in dendritic spines leads to a decrease in network activity [41,42]. Hence, a balance between the host immune response and the parasitic modulators controls the persistence and progression of toxoplasmosis. Direct symptoms of chronic toxoplasmosis are not fully unraveled, and most published studies correlate this disease status with neuropathies with only little molecular proof [43,44] (see sections below). Yet, the reactivation of chronic toxoplasmosis following immunosuppression is frequently reported and may lead to dire consequences reaching death. 

### 2.2. Toxoplasmosis in Immunocompromised Patients

The host immune response plays a key role in the control of parasite replication and maintenance of tissue cysts. With the growing number of individuals receiving immune-suppressive therapies, clinicians are aware of the potential occurrence of *Toxoplasma* encephalitis, not only during the reactivation of latent infection, but also as a primary infection [45]. Indeed, despite the availability of prophylactic and treatment options, the reactivation of chronic toxoplasmosis still occurs and can become life threatening [4,46,47,48]. In immunocompromised patients, the reactivation of chronic toxoplasmosis is due to various factors impairing the protective cellular immune response such as HIV infection, immunosuppressive therapies administered in the context of hematopoietic stem cell transplantation, solid organ transplant, or chemotherapy against cancer [30,49,50,51,52,53,54]. In HIV patients, toxoplasmic encephalitis is the predominant manifestation of the disease, while pulmonary or disseminated toxoplasmosis is more characteristic of transplant patients [2,55]. These patients present with neurologic symptoms, most frequently diffuse encephalopathy, meningoencephalitis, cerebral mass lesions, headaches, confusion, poor coordination, and seizures. Moreover, in patients with HIV, an association between CD4 counts and the prevalence of *T. gondii*-related neurologic symptoms was reported [47]. In that sense, the reactivation of chronic toxoplasmosis becomes a concern when the CD4 count falls below 200 cells/microliter [47]. This reactivation is due to the consequential decrease in IFN-γ and cytokine production, leading to impaired cytotoxic T-lymphocyte activity. Recent data revealed that HIV patients who presented with symptoms of fever and dizziness as part of their *Toxoplasma* encephalitis prodrome sought medical care quicker than those who did not present with these symptoms, leading to the swift administration of treatment, thus reducing mortality [56]. 

The reactivation of chronic toxoplasmosis was also reported following chemotherapy administration. Indeed, several cases of reactivation of cerebral toxoplasmosis following rituximab therapy were described [57,58,59,60]. The reactivation of toxoplasmosis is also a concern in solid organ transplant recipients, either as a manifestation derived from an infected donor, a reactivation of chronic toxoplasmosis in the recipient, or to a much lesser extent, a primary acquired infection following transplantation. The highest risk of toxoplasmosis was described in orthotopic heart transplant recipients due to the propensity of bradyzoite cysts to form in striated muscles. This enhanced the screening for *T. gondii* in these patients prior to transplantation [61]. A retrospective review of solid organ transplant and hematopoietic stem cell transplant recipients with toxoplasmosis between 2002 and 2018 at two large US academic transplant centers was recently conducted. The median time from transplant to toxoplasmosis diagnosis was longer for solid organ transplants than for hematopoietic stem cell transplants, and clinical manifestations were encephalitis (65%), respiratory failure (40%), renal failure (40%), and distributive shock (40%). The cohort 30-day mortality was 45%, and the 90-day mortality was 55% of the cohort [62].

## 3. *Toxoplasma gondii*-Associated Diseases

In healthy individuals, chronic toxoplasmosis was regarded as clinically asymptomatic [63]. However, an increasing number of associations are being made between various medical conditions and *T. gondii* infections [63]. These comprise primary neuropathies, behavioral and psychiatric disorders, and different types of cancer [64].

### 3.1. Toxoplasma gondii and Primary Neuropathies

**Figure 2:**
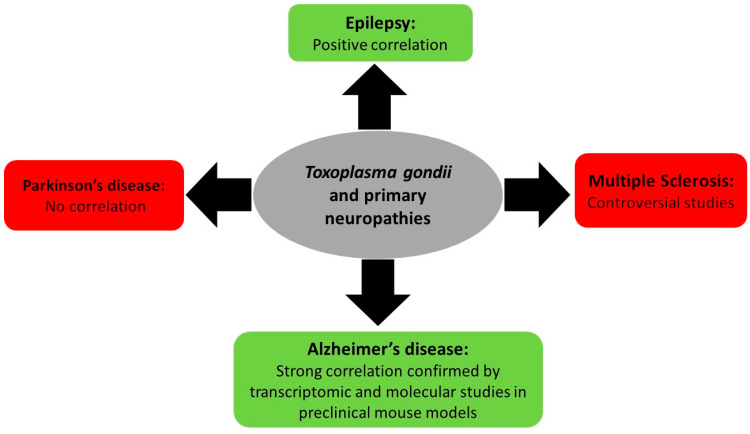
Summary of *Toxoplasma gondii*-associated primary neuropathy diseases and their outcome.

Associations between *T. gondii* infection and primary neurologic diseases such as multiple sclerosis, epilepsy, and Parkinson’s and Alzheimer’s disease remain limited to correlation, controversial studies, and lack a direct molecular proof (Figure 2). In this review, a brief overview of the known literature will be covered.

#### 3.1.1. Toxoplasmosis and Multiple Sclerosis 

Multiple sclerosis (MS) is a chronic autoimmune inflammatory multifactorial disease that affects the nervous system, leading to cognitive, neurological, and physical disabilities. Associations between *T. gondii* and MS relied on data collected from five studies (up to April 2017, 669 MS patients and 770 controls). Four out of five studies showed a negative association between *T. gondii* and MS and only one unveiled a positive association [65]. Another study, which included 164 patients and 481 controls, revealed a negative association between both diseases [66]. More recently, a systematic review including all published articles up to November 2020, which used a random effects model for a global population of 752 MS cases and 1282 controls, added to the controversies. It reported a pooled odds ratio of 0.68 (95% confidence interval = 0.50–0.93), suggesting that toxoplasmosis may play a protective role against MS [67].

#### 3.1.2. Toxoplasmosis and Epilepsy

A systematic literature review used the random effects model on all published articles correlating toxoplasmosis and epilepsy, and showed a calculated odds ratio of 2.25, favoring the association between these two diseases and revealing toxoplasmosis as an epilepsy risk factor [68]. Cryptogenic epilepsies represent 20% of epilepsy syndromes with an unknown etiology that are usually due to a suspected underlying brain disease [69]. A study investigated the correlation between cryptogenic epilepsy and toxoplasmosis by choosing a subpopulation of cryptogenic epilepsy patients and testing for *T. gondii* antibodies. The results were compared with known-cause epilepsy patients and with controls. Cryptogenic epilepsy patients recorded a significant and greater percentage of anti *T. gondii* IgG antibodies (54%) as compared to 22% in known-cause epilepsy patients and 18% in non-epileptic healthy controls [70]. Similarly, ELISA performed on 22 cryptogenic epilepsy patients revealed that 75% of these patients had greater *T. gondii* antibody titers than those recorded among the controls [71]. Finally, a meta-analysis study highlighted the increased odds ratio to 1.72 for *Toxoplasmosis* infection among patients with epilepsy and a significant association between both cryptogenic and active convulsive epilepsy with *T. gondii* infection [72]. These studies favor a potential association between *T. gondii* and epilepsy. 

#### 3.1.3. Toxoplasmosis and Parkinson’s and Alzheimer’s Neuropathies

Antibodies against *Toxoplasma gondii* infection were investigated in Parkinson’s and Alzheimer’s patients. No significant association was reported between toxoplasmosis and Parkinson’s disease [73,74]. Seroprevalence for *T. gondii* was significantly higher in Alzheimer’s patients as compared to their matched controls [75,76,77]. Importantly, Alzheimer’s patients did not witness reactivation of latent toxoplasmosis, with only one case over 105 patients exhibiting positive IgM [78]. Yet, in a meta-analysis on observational studies between *T. gondii* infection and Alzheimer’s disease, only a marginally significant association was noted [73]. At the molecular level, 118 genes (around 27.3%) over 432 susceptibility genes in Alzheimer’s disease are involved in the *T. gondii* host/pathogen interactome [79]. In preclinical models, experiments on BALB/c mice showed that infection with *T. gondii* leads to Alzheimer’s-like symptoms including conflicts in learning and weak memory [80]. In C57BL/6 mice, an accumulation of beta amyloid (Aβ) immunoreactivity and hyperphosphorylated tau, one of the markers of Alzheimer’s disease, was recorded in the brains of mice [81]. Another study reported that *Toxoplasma* infection ameliorates β-amyloidosis in a murine model of Alzheimer’s disease. This was mostly due to the activation and recruitment of monocytes, hence enhancing the degradation of soluble Aβ [82]. The effect of the immunosuppression induced by *T. gondii* infection on the pathophysiology of Alzheimer’s disease was also addressed in a murine model of Alzheimer’s disease (Tg2576). While IFN-γ levels remained unchanged, the levels of anti-inflammatory cytokines were significantly higher in *T. gondii*-infected mice than in uninfected mice. Furthermore, β-amyloid plaque deposition in the cortex and hippocampus was remarkably lower and better cognitive capacities were observed in *T. gondii*-infected mice, demonstrating a positive impact of *T. gondii*-induced immunosuppression on Alzheimer’s progression in a murine model [83]. Given that the progression of Alzheimer’s disease deteriorates upon the accumulation of Aβ plaques, which are eliminated through microglial phagocytosis, the association between microglial proliferation and Aβ plaque burden using brain tissues isolated from an Alzheimer’s disease murine model (5XFAD) following infection with *T. gondii* was studied. In the infected group, a significant decrease in the amyloid plaque burden concurrent with an extensive proliferation of homeostatic microglial proliferation and an increased number of plaque-associated microglia were observed. Hence, it was concluded that chronic *T. gondii* infection can induce microglial proliferation in the brains of mice with progressed Alzheimer’s disease, a promising approach for the treatment of this neuropathy [84]. In conclusion, the relationship of *T. gondii* and the development of Alzheimer’s disease and cognitive impairment require further studies on human subjects and animal models [85] to elucidate the possible role of toxoplasmosis in the etiology of Alzheimer’s disease.

### 3.2. Toxoplasma gondii, Psychiatric and Behavioral Disorders

**Figure 3:**
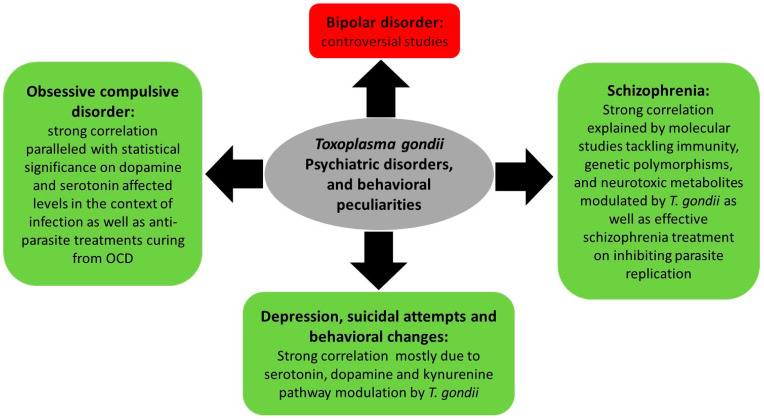
Summary of *Toxoplasma gondii*-associated psychiatric and behavioral disorders and the molecular status dictating these associations.

One of the mechanisms ensuring *T. gondii* expansion throughout its life cycle involves behavioral changes between intermediate and final hosts. Indeed, behavioral peculiarities were reported in infected rodents, which exhibit attenuated aversion and fear and do not flee cats’ urine odor (reviewed in [86]). In humans, an increasing body of literature indicates that chronic toxoplasmosis is associated with aberrant host behavior [87] and influences the progression of psychiatric disorders [88], such as schizophrenia, bipolar disorder, and obsessive compulsive disorder [89,90] (Figure 3). This is partly due to altered dopamine levels following *T. gondii* infection [91,92,93,94]. The mechanisms underpinning these changes are still vague and complex, and seem to involve the immune response, hormonal changes, genetic and epigenetic factors as well as structural effects on the infected area of the brain.

#### 3.2.1. Toxoplasmosis, Depression, and Behavioral Changes

Depression, a mood disorder [95], is characterized by altered levels of serotonin and dopamine. Decreased levels of serotonin are at the cornerstone of depression. Tryptophan, serotonin’s precursor, is essential for *Toxoplasma* growth [96,97]. *T. gondii* infection triggers inflammatory molecules such as IL-2, IFN-γ, and TNF-α, which consequently upregulate IDO and TDO, hence shunting tryptophan into a degradation pathway. Tryptophan is degraded into kynurenine by indoleamine-2,3-dioxygenase (IDO) and tryptophan-2,3-dioxygenase (TDO). The depletion of tryptophan promotes the onset of depression [98,99,100,101].

The kynurenine pathway is known to produce neurotoxic metabolites such as kynurenic acid (KYNA), quinolinic acid (QUIN), and 3-hydroxykynurenine. This pathway is activated during *Toxoplasma* infection, and abnormal levels of KYNA were reported in preclinical mouse models of *T. gondii* as well as in subjects infected with this parasite [102,103]. While abnormal levels of KYNA were shown to decrease both dopamine and glutamate extracellular concentrations in rodent models [104], *T. gondii* infection does not seem to exhibit same effect. Indeed, it was reported that *T. gondii tyrosine* and *phenylalanine hydroxylase* genes catalyze tyrosine and phenylalanine, both of which are precursors of dopamine [105], which may lead to increased dopamine levels and presumably less depression symptoms. In humans, studies demonstrated that chronic toxoplasmosis is associated with systematic changes in human personality [106]. A study enrolling 285 participants revealed that depressed individuals who attempted suicide exhibited higher *T. gondii* IgG titers than those who did not [107]. Elderly women (aged 60 or above) are more prone to suicidal attempts when seropositive for *T. gondii* [108]. A detailed review involving three wide meta-analyses in different European countries on the associations between *T. gondii* serology and suicidal behavior reported a 39 to 57% elevation of odds of suicide attempts in *T. gondii* IgG-positive patients [109]. One case report showed that a depressed 32-year-old male did not respond to antidepressant therapy until he was treated for acute toxoplasmosis, suggesting a probable association between toxoplasmosis and depression [110].

#### 3.2.2. Toxoplasmosis and Schizophrenia

Schizophrenia is a psychiatric disorder encompassing varying degrees of delusions, disorganized thoughts, hallucinations that are mainly auditory, disorganized behaviors, and negative symptoms such as having a blunted affect. Difficulties in social interactions, emotions, and overall functionality are also noticed. Different studies associated T. gondii infections with schizophrenia. It was indeed reported that chronic toxoplasmosis associated with schizophrenia is characterized by a significant reduction in gray matter; a finding not seen in the control groups [111]. Different studies investigated the association between toxoplasmosis and schizophrenia. The first meta-analysis in that regard was performed in 2007 and updated 5 years later [112,113], and revealed significantly elevated seropositive rates of anti-*Toxoplasma* IgG and IgM in schizophrenic patients. Similar results were obtained on around 800 Chinese patients with schizophrenia as compared to their matching controls [114], and in a large case–control study on around 80,000 individuals, where *T. gondii* IgG titers were detected in the plasma samples of 25% of patients and were significantly associated with schizophrenia [115]. Genetic susceptibility is one of the main risk factors to develop schizophrenia. It was reported that people infected with *T. gondii* and suffering from schizophrenia have polymorphisms in genes encoding glucocorticoid-inducible kinase 1 (SGK1) and solute carrier family 2 member 12 (SLC2A12), supporting the plausible role of inflammatory processes and infections as risk factors for psychotic behaviors [116], but these associations did not achieve statistical significance on a genome-wide level [116]. Moreover, *T. gondii* infection represents a risk factor to develop schizophrenia in susceptible individuals or to exacerbate disease progression, but *T. gondii* alone does not trigger schizophrenia, leading to hippocampal pathologies and increased KYNA levels, which decrease dopamine and glutamate, thus altering cognitive functions [101], and leading to the production of neurotoxic metabolites such as quinolinic acid and 3-hydroxykynurenine [117,118]. At the molecular level, a major mental illness-related susceptibility factor, the “Disrupted in schizophrenia” (DISC1), is involved in host immune responses against *T. gondii* infection, and certain genotypes of DISC1, particularly the 607 Phe/Phe, correlate with higher serology against this parasite [119]. Furthermore, decreased CD8^+^ T activity and loss of their proliferation and cytokine secretion potentially increase the risk of schizophrenia as a result of focal necrosis and inflammation. It is important to recall that CD8^+^ T cell numbers, which play a crucial role in fighting *T. gondii* infections, are decreased in schizophrenic patients [120,121]. Finally, some medications used to treat schizophrenia inhibited the replication of *T. gondii* in cell culture [122].

#### 3.2.3. Toxoplasmosis and Bipolar Disorder

Bipolar disorder (BD), known as manic depression, is a psychiatric disorder in which the patient suffers from rapid or sudden mood changes fluctuating between extreme euphoria to extreme sadness and depression. The etiology of BD is complex and encompasses brain and peripheral chronic inflammation, immune dysfunction, genetic inheritance, and environmental risk factors. Different correlation studies were conducted between toxoplasmosis and bipolar disorders and were contentious. While some studies revealed an increased prevalence of *T. gondii* in these individuals [123,124,125,126], other studies showed no correlation [127,128]. In BD patients infected with *T. gondii*, increased levels of kynurenine and kynurenic acid are documented, which correlates with fluctuating levels of dopamine and glutamate as well as the production of neurotoxic factors [104].

#### 3.2.4. Toxoplasmosis and Obsessive Compulsive Disorder

According to the World Health Organization, obsessive compulsive disorder (OCD) is a mental disorder ranked among the top ten life-quality-reducing mental disorders. People with OCD cannot control their thoughts and obsessively repeat activities such as washing hands, checking doors, among others. A meta-analysis pooling 11 studies (9873 participants, including 389 OCD patients) showed a strong correlation between the prevalence of toxoplasmosis and OCD, with a statistically significant odds ratio of correlation with increased dopamine levels [129]. Other studies suggest that toxoplasmosis leads to changes in hypothalamic–pituitary–adrenal gland axis activity and hormonal disorders including serotonin, which can also lead to OCD [130]. The treatment of two children diagnosed with OCD and seropositive for *T. gondii* with anti-protozoan medication resulted in both decreased levels of antibodies and a total cure from OCD [131].

### 3.3. Toxoplasma gondii and Cancers: Modulation of miRNAs as One Molecular Explanation of Toxoplasma-Associated Brain Cancers

Different types of cancers, especially brain cancers, are associated with *T. gondii* infection. Indeed, the incidence of adult brain cancers is higher in countries with common infection with *T. gondii* [132,133,134,135]. This positive correlation was associated with the ability of the parasite to interfere with the brain cells’ miRNAome [135], which might lead to brain tumor development [136]. Beyond brain cancers, higher anti-*T. gondii* antibodies were observed in various types of cancer including lung, prostate, cervix, and endometrial cancers [134,137,138,139]. 

*T. gondii* alters the expression of crucial miRNAs responsible for mounting an immune response in the host cell against the infection [140]. These miRNAs target many transcripts associated with immune functions, such as cytokines, chemokines, and interleukins. Studies have focused on global host miRNA responses following *T. gondii* infection in multiple cell types such as human foreskin fibroblasts (HFFs) and neuro-epithelial cells in various regions in the body such as the brain, plasma, spleen, and liver. The miR-17–92 cluster, one of the upregulated miRNAs during infection [141], inhibits host cell apoptosis, a survival strategy of *Toxoplasma* [142,143]. miR-132 is a small endogenous cyclic AMP-responsive element binding (CREB)-regulated miRNA known to have both immune and neural functions. Several neurological disorders were associated with the dysregulation of miR-132, including *Toxoplasma*-induced encephalopathy, Alzheimer’s disease, Parkinson’s disease, epilepsy, depression, and schizophrenia [144]. Likewise, infected neuro-epithelioma cells with different types of *T. gondii* strains showed an upregulation of miR-132 involved in the signaling of dopamine receptors by more than two-fold [145]. 

In humans, several studies validated the expression of miRNA in brain tumors including glioblastoma, pituitary adenoma, and medulloblastoma compared to other tissues [146,147,148]. Ryan et al.’s studies showed that in the case of meningioma, there is a noticeable level of antibodies for *Toxoplasma* [133]. A meta-analysis study including 37 countries showed that in areas of high prevalence of *Toxoplasma* infection, there is approximately a two-fold increase in brain cancer risk, suggesting the association between *Toxoplasma* occurrence and adult brain cancers [134]. Another epidemiological study revealed that rates of death in brain cancer are positively correlated with sero-prevalence for *Toxoplasma*, especially for people who are aged 55 years or older [149]. Primary human astrocytic glioma tissue specimens over-express the miR-17–92 cluster compared to non-neoplastic brain control tissues [150,151]. During infection, a decrease in phosphatase and tensin homolog (PTEN) in brain cells by miR-17–92 activated the AKT pathway, which promotes survival and growth in response to extracellular signals, resulting in brain cancer development [152]. Considering the significance of miRNAs in the development of brain carcinogenesis, it is worth noting that the *Toxoplasma* genome codes for ostentatious RNA silencing machinery and endogenous small silencing RNAs, including miRNAs [153].

## 4. Current Treatments

The treatment of toxoplasmosis remains limited to relatively general anti-parasitic/anti-bacterial drugs (reviewed [154,155]). These include spiramycin, azithromycin, atovaquone, pyrimethamine–sulfadiazine, pyrimethamine–clindamycin, and trimethoprim–sulfamethoxazole. Unlike its mammalian host, *T. gondii* is unable to use preformed dietary folates and has to synthesize folates *de novo* [156]. Thus, the recommended first-line therapy remains the synergistic combination of pyrimethamine, an inhibitor of dihydrofolate reductase (DHFR) enzyme, and sulfadiazine, an inhibitor of dihydropteroate synthase [156,157,158]. This combination is usually administered with folinic acid (leucovorin) to reduce harmful side effects, amongst which is bone marrow suppression [159]. The aforementioned combination blocks the biosynthesis of parasitic folate, thus interrupting nucleic acid synthesis and parasite replication. However, this combination is associated with several limitations, including but not limited to hematological side effects such as neutropenia, thrombocytopenia, and leukopenia [160]. Other side effects include an elevation in serum creatinine and serum liver enzymes, hypersensitivity or allergic reactions [161], and the emergence of resistant strains [162,163], especially in immunocompromised patients [164]. Pyrimethamine can potentially be teratogenic and should not be used during the early months of pregnancy [164]. These drugs, whether given prophylactically or therapeutically, target only the acute phase of the infection and remain useless against the chronic form of toxoplasmosis represented by tissue cysts [165]. To date, there is no approved therapy that eliminates the tissue cysts responsible for the chronic stage of *Toxoplasma* infection [162,166,167]. Degerli et al. evaluated the effectiveness of azithromycin, a protein synthesis inhibitor, in a preclinical mouse model infected with *T. gondii* tachyzoites and following the development of bradyzoite stages. The study showed that the drug is effective both prophylactically and during the early stages of infection [168]. In pregnant women, treatment is based on the administration of spiramycin or sulfadiazine–pyrimethamine–folinic acid (SPFA) if fetal infection is confirmed [168].

In a systematic review, Montazeri et al. evaluated the in vitro and in vivo activities of anti-*Toxoplasma* drugs and compounds during the time period between 2006 and 2016. Eighty clinically available drugs and a large number of new compounds with more than forty mechanisms of action were summarized. Several target-based drug screens were also identified, including mitochondrial electron transport chain, calcium-dependent protein kinase 1, type II fatty acid synthesis, DNA synthesis, and DNA replication, among several others [167]. Most of these drugs are effective against tachyzoites, and only very few trigger bradyzoites or the back and forth switch between both stages [167]. It is worth noting that an ideal drug against toxoplasmosis should not only be effective against the proliferative tachyzoite stage of the parasite but also against the tissue cyst stage, particularly since the chronic form is the most prevalent among all diseases caused by and associated with this parasitic infection. In addition, these drugs should be able to cross the blood brain barrier and penetrate the brain, where the accumulation of bradyzoite cysts is high [169]. Recently, the effect of imiquimod, an efficient immunomodulatory drug, was explored in murine models of acute and chronic toxoplasmosis. Treatment with imiquimod during acute toxoplasmosis reduced the number of brain cysts while rendering the remaining ones un-infectious. Importantly, treatment with imiquimod, post-establishment of CT, significantly reduces the number of brain cysts, leading to a delay or abortion of reactivation. Molecularly, imiquimod upregulated the expression of Toll-like receptors and activated the MyD88 pathway, resulting in the induction of the immune response to control reactivation [170].

## 5. Toxoplasmosis and Prophylaxis: Available and Potential Vaccine Strategies

Due to the burdensome effects of toxoplasmosis and the failure and/or adverse effects of the currently used therapeutic approaches, several attempts were made to develop vaccines against *T. gondii* (for a review, see [171]). In 1995, the first commercial vaccine for toxoplasmosis, Ovilis Toxovax, was developed. It consisted of an injectable suspension of attenuated parasites of the strain S48, originally isolated from a case of ovine abortion in New Zealand. Following approximately 3000 passages in mice, this strain lost its ability to differentiate into tissue cysts in mice and into oocysts in cats [172,173]. This live vaccine was used to prevent toxoplasmosis-induced abortions in sheep, but did not reach human trials due to the high capacity of the parasite to revert back to its pathogenic features. Other vaccine candidates were tested, including apical complex proteins from *T. gondii* (rhoptries, micronemes, and dense granules), multi-antigen vaccines, and other adjuvants [174,175]. However, these attempts failed to yield proper protection against toxoplasmosis in humans [176]. In addition, some classes of antigens were proposed to be potential vaccine candidates. These include the Recombinant Surface Antigen-1 (SAG-1), which is a GPI-anchored and highly immunogenic surface marker of the tachyzoite stage of *T. gondii* and which may protect against acute toxoplasmosis [177,178] and thus brain cyst formation [179,180]. Recombinant GRA4 and ROP2 given with Alum adjuvant were also proposed and provided protection against brain cyst formation in C57BL/6 mice [181]. A mixture of SAG1, GRA1, and Merozoite Antigen-1 (MAG1), given with Freund’s Complete Adjuvant, reduced brain cyst burden by 90% in BALB/c mice. A mixture of GERBU, an adjuvant based on cationic lipid solid nanoparticles and Nacetylglucosaminyl- N-acetylmuramyl-l-alanyl-d-isoglutamine, a glycopeptide derived from *Lactobacillus bulgaricus* cell walls, with GRA7 and a MIC2-MIC3-SAG1 chimeric protein provided an 80% reduction in brain cysts in outbred SWISS mice following challenge with *T. gondii* 76K [182,183]. Finally, the double knock-out of MIC1-MIC3 genes markedly impaired virulence and conferred protection from *T. gondii* [184].

An ideal vaccine must possess different antigens from all three infective stages of *T. gondii* to increase the ability of inducing a strong immune response [185]. Vaccine development proposed multi-epitope DNA vaccines composed of CD8^+^ T cell-eliciting, rhomboid protease 4 and GRA14 of the RH strain, as well as CD4^+^ helper T lymphocyte epitope(s) administered with lipid adjuvant [184], coated with calcium phosphate nanoparticles [186] or recombinant proteins formulated in Poly (DL-lactide-co-glycolide) microspheres [187], or virus-like particles (VLPs) [188]. These multi-epitope vaccine attempts increased both the cellular and humoral responses by the augmentation of memory CD8^+^ T cells, thus inducing a higher IFN-γ production, and protected mice against parasite burden when challenged with *T. gondii*.

Altogether, and beyond the Toxovax vaccine used on sheep, these studies remain limited to rodents and require more investigation to come up with an ideal vaccine of *Toxoplasma* for clinical trials.

## 6. Concluding Remarks

Despite its prevalence, toxoplasmosis remains a neglected disease. Increased statistical correlations between toxoplasmosis and neurologic, psychiatric, and cancer disorders have been unveiled. Addressing the molecular players underlying these associations is paramount in creating avenues for new treatment modalities, especially in light of the absence of a gold standard treatment and a human vaccine against toxoplasmosis. To reach this aim, establishing appropriate animal models of primary neuropathies and behavioral disorders is a must. Indeed, some available pre-clinical models recapitulate the features of some of these diseases, but the absence of the appropriate model remains a challenge for most of them. The recent advances in high-throughput sequencing and proteomics techniques should help in apprehending the correct molecular markers and biomarkers between the parasite and its associated diseases. In addition, the ease of genetic manipulation using multiple tools, including the CRISPR-cas9 targeted disruption or knock-in for genes in *T. gondii*, will help increase our understanding of the molecular players to confirm the positive correlations between toxoplasmosis and primary neuropathies/associated diseases and solve the enigma of the available controversial studies. Finally, clinicians should increase their awareness of reactivation in immunocompromised patients, an area of interest in which quick molecular diagnostic tests are still lacking.

## Data Availability

Not applicable.
